# Recurrence of breech presentation in consecutive pregnancies

**DOI:** 10.1111/j.1471-0528.2010.02576.x

**Published:** 2010-06

**Authors:** JB Ford, CL Roberts, N Nassar, W Giles, JM Morris

**Affiliations:** a Kolling Institute of Medical Research, University of SydneyAustralia; b Royal North Shore HospitalSydney, Australia

**Keywords:** Breech presentation, record linkage, recurrence risk

## Abstract

**Objective:**

To investigate the recurrence risk of breech presentation at term, and to assess the risk factors that contribute to its recurrence.

**Design:**

Cohort study.

**Setting:**

New South Wales, Australia.

**Population:**

Women with their first two (*n* = 113 854) and first three (*n* = 21 690) consecutive singleton term pregnancies, in the period 1994–2002.

**Methods:**

Descriptive statistics including rates, relative risks and adjusted relative risks, as determined from logistic regression and Poisson analyses.

**Main outcome measures:**

Rates and risks of occurrence and recurrence of breech presentation at birth in each pregnancy, and maternal and infant risk factors associated with breech recurrence.

**Results:**

First-time breech presentation at term occurred in 4.2% of first pregnancy deliveries, 2.2% of second pregnancies and 1.9% of third pregnancies. The rate of breech recurrence in a second consecutive pregnancy was 9.9%, and in a third consecutive pregnancy (after two prior breech deliveries) was 27.5%. The relative risk of breech recurrence in a second pregnancy was 3.2 (95% CI 2.8–3.6), and in a third consecutive breech pregnancy was 13.9 (95% CI 8.8–22.1). First pregnancy factors associated with recurrence included placenta praevia [adjusted relative risk (aRR) 2.2; 95% CI 1.3–3.7], maternal diabetes (aRR 1.4; 95% CI 1.0–2.1) and a maternal age of ≥35 years (aRR 1.2; 95% CI 0.9–1.6). Second pregnancy factors included birth defects (aRR 2.5; 95% CI 1.4–4.2), placenta praevia (aRR 2.5; 95% CI 1.5–4.1) and a female infant (aRR 1.2; 95% CI 1.0–1.5).

**Conclusions:**

The increased recurrence risk of breech presentations suggests that women with a history of breech delivery should be closely monitored in the latter stages of pregnancy.

## Introduction

Breech presentation has an increased risk of neonatal mortality compared with the overall birthing population.^1^ Much attention has been focussed on the optimal mode of delivery for breech-presenting babies. Following the findings of the Term Breech Trial, of fewer adverse outcomes among those delivered by planned caesarean section than by planned vaginal delivery,^2^ birth is now more likely to occur by caesarean section.^3^ Regardless of mode of delivery, there are increased risks of adverse maternal or neonatal outcomes associated with breech presentation.^4^,^5^

Although studies have investigated risk factors for breech birth at term,^6^,^7^ few have identified predictive factors of breech presentation in a second pregnancy. Women with a prior caesarean delivery are at increased risk for malpresentation at their second delivery (compared with primary vaginal deliveries at first delivery).^8^ A Danish study reported that 15.1% of second births were breech presentation, with higher rates after primary caesareans.^9^ However, these studies did not account for the indication for primary caesarean deliveries, which could have been malpresentation.

Similarly, there are very few studies investigating recurrence risk of breech presentation.^1^,^10^,^11^ Women (and their caregivers) are interested in the future reproductive consequences of breech presentation. Research on recurrent pregnancy outcomes allows clinicians to provide appropriate counselling, and to guide the management of patients with a history of pregnancy complications.^12^,^13^ This paper uses cross-sectional and longitudinally linked birth, hospital and birth defect data sets to determine population-based recurrence rates and risks, as well as identifying the factors in a first or second pregnancy that increase the likelihood of another breech-presenting infant.

### Aims

We aim to: (i) evaluate recurrence risks for breech presentation at term, and (ii) assess risk factors that contribute to the recurrence.

## Methods

### Data sources

The study population included all 699 982 women having singleton term births in New South Wales between 1994 and 2002. One-third of the Australian population (∼7 million people) reside in New South Wales (NSW), with 90 000 births per annum.^14^,^15^ Data were obtained from population-based birth, hospital discharge and birth defects registry records that were probabilistically linked and de-identified for analysis, using methods that have been described previously.^6^,^16^,^17^ Birth data are from the Midwives Data Collection, a legislated population-based surveillance system covering births at ≥20 weeks of gestation or with ≥400 g birthweight. Information on maternal characteristics, pregnancy, labour, delivery and infant outcomes are recorded by the attending midwife or doctor. Hospital discharge data are from the Admitted Patients Data Collection, a census of all NSW inpatient hospital discharges (public and private), with diagnoses and procedures coded for each admission based on information from the medical records, according to the ninth and tenth revision of the International Statistical Classification of Diseases and Related Health Problems (ICD-9CM and ICD10AM). Over the study period the number of possible fields for recording diagnoses increased from 11 to 40; however, for consistency over time only diagnoses reported in the first 11 fields were included in this study. The NSW Birth Defects Register is a population-based surveillance system established to monitor major birth defects diagnosed during pregnancy, at birth, or up to 1 year of age.^15^ Birth defects include any structural defects such as anencephaly, hypospadias and gastroschisis, and exclude birth injuries and minor anomalies such as skin tags, positional talipes, birthmarks or unstable hips.^15^ Birth defects were then classified according to body system and major category of defects.

Breech presentation was identified in the Midwives Data Collection by a tick box recording presentation at birth. Two validation studies of presentation recording (against medical records) demonstrated high levels of agreement for presentation at birth (98.3 and 98.5%), with kappa results of 0.84 and 0.87.^18^,^19^ Maternal age, infant sex, birthweight for gestational age, mode of delivery, place of delivery and maternal smoking were identified from birth data, whereas maternal diabetes and placenta praevia were identified from hospital data, and maternal hypertension was identified from either birth or hospital data. The choice of data set for ascertaining risk factors was based on validation study results indicating the most accurate sources.^19^–^21^ Birth defects diagnosed during pregnancy or at birth were identified via the Birth Defects Register.

### Analysis

We determined the rate of the first occurrence of breech presentation at term in first, second or third pregnancies, and the recurrence rates for women with a history of breech presentation at birth in their first and/or second pregnancies, using contingency table analysis. Analysis was restricted to term breech deliveries (≥37 weeks of gestation), thereby excluding 11 441 deliveries. Women with a first delivery prior to 1994, or with pregnancies that were not consecutive, or with parity data missing for any pregnancy, were excluded. Log–binomial models were used to estimate relative risks and confidence intervals.^22^ Where models didn’t converge, log–Poisson models were used, as they provide a consistent, but not fully efficient, estimate of the relative risk and its confidence intervals.^23^ For multivariate risk factor analyses, all variables with a crude association of *P* < 0.1 were included. Recurrence rates were expressed as rates and crude relative risks, whereas multivariate results were expressed as adjusted relative risks. Adjusted relative risks were only calculated for the risk of recurrence in a second pregnancy, and not for a third pregnancy, given the small event rate and the number of adjustment factors.^24^

As breech presentation is more likely to occur among pregnancies with infant birth defects or placenta praevia, sensitivity analyses were conducted to assess the impact of recurrence with and without these conditions. Breech occurrence and recurrence rates by mode of delivery and place of delivery were also investigated. The study was approved by the University of Sydney Ethics Committee (02-2008/10674).

## Results

There were 113 854 women with at least a singleton first and second birth at ≥37 weeks of gestation in the period 1994–2002. Of these women, 21 690 had at least three consecutive singleton pregnancies.

**Figure 1:**
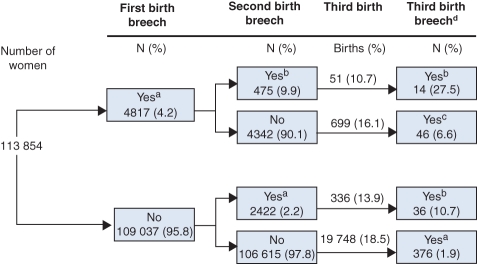
Breech occurrence and recurrence for term, singleton births in the period 1994–2002 in New South Wales. Note: all first pregnancy records have a second pregnancy recorded, but not all second pregnancy records have a third pregnancy recorded; ^a^occurrence; ^b^recurrence; ^c^recurrence with an intervening uneventful pregnancy; ^d^proportions are calculated based on women who went on to have a third pregnancy.

Among all births in New South Wales in the period 1994–2002 there was no significant trend (*P* = 0.11) in term births with breech presentation, with an overall rate of 3.4% of deliveries. First occurrence of a term breech delivery was highest in first pregnancies (4.2 per 100 births), dropping to 2.2 and 1.9 per 100 births, respectively, at second or third pregnancy (Figure 1; Table 1). The overall rate of occurrence of breech presentation was 2.5 per 100 births in a second pregnancy, and 2.2 per 100 births in a third pregnancy. There were no statistically significant changes in the frequency of breech presentation by parity during the study period.

**Table 1:** Rate of breech presentation at term in the first, second and third pregnancies in the period 1994–2002 in New South Wales

Pregnancy	All pregnancies			Women with no previous breech presentation		
	Breech presentation at term			Breech presentation at term		
	Total number of births	Number of cases	Rate per 100 births	Total number of births	Number of cases	Rate per 100 births
First	113 854	4817	4.23	113 854	4817	4.23
Second	113 854	2897	2.54	109 037	2422	2.22
Third	21 690	472	2.18	19 748	376	1.93

After one breech delivery, the recurrence rate for a second pregnancy with breech presentation was 9.9%, and the recurrence rate was 27.5% for a third consecutive breech pregnancy (Figure 1; Table 2). The second breech delivery rate was similar, irrespective of whether the first occurrence was in a first or second delivery (breech presentation recurred after 9.9% of first delivery breech presentations, and after 10.7% of second delivery breeches). There was no difference in the rates of breech occurrence and recurrence when mothers diagnosed with placenta praevia at either pregnancy were excluded, or when babies with birth defects were excluded (data not shown).

**Table 2:** Risk recurrence of breech presentation at term amongst the first three pregnancies in the period 1994–2002 in New South Wales

Presentation at term in each birth			Breech risk in latest birth (%)	Relative risk (95% confidence interval)	
First	Second	Third		Unadjusted	Adjusted*
Vertex	Breech	—	2422 (2.2)	1.00 (reference)	1.00 (reference)
Breech	Breech	—	475 (9.9)	4.44 (4.04–4.88)	3.18 (2.83–3.56)
Vertex	Vertex	Breech	376 (1.9)	1.00 (reference)	—
Vertex	Breech	Breech	36 (10.7)	5.54 (4.00–7.67)	—
Breech	Vertex	Breech	46 (6.6)	3.60 (2.70–4.80)	—
Breech	Breech	Breech	14 (27.5)	13.90 (8.75–22.09)	—

* Analyses adjusted for the risk factors presented in Table 3.

Given the small numbers of third breech deliveries and the number of factors for adjustment, adjusted relative risks have only been calculated for recurrence of breech presentation in a second pregnancy.

**Table 3:** Risk factors for a recurrent breech presentation at term in a second pregnancy in the period 1994–2002 in New South Wales

Factor	Second delivery, *n* (%)		Crude RR (95% CI)	Adjusted RR (95% CI)*
**First pregnancy factors**	No breech *n*= 4342 (90.1)	Breech *n* = 475 (9.9)		
**Maternal age**				
<20 years	261 (6.0)	16 (3.4)	0.59 (0.36–0.95)	0.57 (0.34–0.95)
20–34 years	3739 (86.1)	108 (85.9)	Reference	Reference
≥35 years	342 (7.9)	51 (10.7)	1.32 (1.00–1.73)	1.23 (0.93–1.63)
**Maternal diabetes**	156 (3.6)	26 (5.5)	1.47 (1.02–2.13)	1.45 (1.00–2.09)
**Placenta praevia**	34 (0.8)	12 (2.5)	2.69 (1.64–4.41)	2.23 (1.35–3.68)
**Caesarean delivery**	3732 (86.0)	422 (88.8)	1.27 (0.97–1.67)	1.19 (0.90–1.57)
**Baby birthweight for gestational age**				
<10th percentile	572 (13.2)	69 (14.5)	1.13 (0.89–1.45)	1.17 (0.92–1.50)
10–90th percentile	3471 (80.0)	364 (76.6)	Reference	Reference
>90th percentile	298 (6.9)	42 (8.8)	1.30 (0.96–1.76)	1.26 (0.93–1.70)
**Second pregnancy factors**				
**Placenta praevia**	27 (0.6)	11 (2.3)	2.98 (1.80–4.94)	2.48 (1.49–4.11)
**Delivery hospital**				
Rural/district	1347 (31.0)	149 (31.4)	1.12 (0.91–1.38)	1.16 (0.94–1.43)
Regional/tertiary	1747 (40.2)	171 (36.0)	Reference	Reference
Private	1248 (28.7)	155 (32.6)	1.24 (1.01–1.52)	1.15 (0.94–1.42)
**Female infant**	2058 (47.4)	248 (52.2)	1.23 (1.02–1.49)	1.22 (1.03–1.45)
**Birth defect**	35 (0.8)	10 (2.1)	2.28 (1.31–3.97)	2.41 (1.39–4.19)

* Analyses adjusted for all other factors presented.

The crude relative risk of breech recurrence in a second pregnancy was 4.4 (95% CI 4.0–4.9), and in a third consecutive breech pregnancy was 13.9 (95% CI 8.8–22.1) (Table 2). The adjusted relative risk for breech recurrence in a second pregnancy was 3.2 (95% CI 2.8–3.6) (Table 2).

First pregnancy factors associated with subsequent breech presentation in a second pregnancy were placenta praevia, maternal diabetes, baby birthweight for gestational age, maternal age of 35 years or over and caesarean delivery (Table 3). Second pregnancy factors associated with a second breech presentation were birth defects, placenta praevia, female babies and delivery hospital (Table 3). Other factors investigated, but which showed no crude association with second breech presentation, included maternal hypertension in either pregnancy, maternal smoking, first female baby, first baby with a birth defect and birth interval. The most commonly reported birth defects among deliveries with breech presentation were musculoskeletal, chromosomal and cardiovascular defects.

Fourteen percent of first pregnancies with breech-presenting infants delivered vaginally. Among women with a first breech presentation in a second pregnancy, 23% delivered vaginally. Following a first vaginal breech delivery, 54% of second breech deliveries occurred vaginally. Following a first breech delivery by caesarean, 99% of second breech deliveries were caesareans. The majority of first breech caesarean deliveries did not involve labour (70.4%): of these, 40.0% delivered at 37–38 weeks of gestation, and 60.0% delivered at 39 weeks of gestation or later. Fifteen percent of second breech deliveries involved a caesarean with labour, 78% were caesareans without labour and 7% were vaginal breech deliveries.

Rates of first breech presentation at tertiary and other public hospitals were similar, at around 4.0%, with a slightly higher proportion of first breech presentations among private hospital births (5.3%, *P* < 0.0001). There was no significant difference between rates of second breech presentation at tertiary, other public and private hospitals.

## Discussion

Overall, the rate of breech occurrence in a first pregnancy (4.2%) is almost double the rate of occurrence in a second (2.2%) or third pregnancy (1.9%). Women are at an increased risk of breech recurrence after a breech presentation in a previous pregnancy. Whereas one in 20 women are likely to have a first breech presentation in a first pregnancy, one in ten will have a breech-presenting baby in a subsequent pregnancy, and in a third pregnancy the rate increases even further to one in four. An intervening cephalic delivery decreases, but does not totally ameliorate, the risk. Women with a history of breech presentation have a three-fold increased risk in a second pregnancy (adjusted RR 3.2; 95% CI 2.8–3.6), and an up to 14-fold increased risk in a third pregnancy (RR 13.9; 95% CI 3.8–22.1).

There is very little longitudinally linked, population-based birth data available that can be used to determine recurrence risk. The recurrence rates and risks reported in our study are similar to the only other population-based study reporting breech recurrence risk. Albrechtsen *et al.*,^25^ using birth registry data from 1967 to 1994, reported breech recurrence rates of 8.9 and 21.4%. The authors concluded that the high risk of recurrence suggests the effects of recurring specific causal factors of genetic or environmental origin; however, they did not investigate the factors associated with recurrence.

To our knowledge this is the first population-based study to investigate risk factors associated with breech recurrence. Our analysis of risk factors identified that placenta praevia in a first or second pregnancy and/or birth defect in a second pregnancy were the most significant risk factors for breech recurrence. With the exception of birthweight for gestational age, it was maternal factors from the first birth that were associated with recurrence, including: maternal age, placenta praevia and maternal diabetes. This suggests that persistent maternal factors rather than first pregnancy fetal or infant factors play a role in repeat breech presentation.

External cephalic version (ECV) for breech presentation at term is an effective means of reducing non-cephalic presentation and caesarean section, with a systematic review finding an overall ECV success rate of 68% (65–70%).^26^ Although we cannot identify women who have undergone ECV, there is a lower rate of breech presentation in tertiary and large regional hospitals, despite the higher proportion of women with risk factors for breech presentation delivering at these hospitals. This suggests that these hospitals may be successfully undertaking ECV for first breech presentation. The lack of a difference between hospital rates of second breech presentation is not surprising, as a scarred uterus (the likely outcome from a first breech presentation) is a relative contraindication to performing ECV.^27^,^28^

Mode of delivery is an important consideration in any pregnancy diagnosed with breech presentation. This study was unable to determine the proportion of antenatally diagnosed breech pregnancies; however, 30% of women with a first breech presentation underwent caesarean deliveries after labour. This is comparable with the 25% of women reported to have undiagnosed breech in another study of singleton term pregnancies.^29^

Fifteen percent of second breech deliveries were caesareans after labour. Australian birth data cannot identify the intended mode of delivery; however, findings from a state-wide review of medical records found that among women with a caesarean section after the onset of labour, 16% were elective/planned caesareans with labour before the planned date.^30^ It is likely that the majority of the second breech caesarean deliveries after labour in our study represent unplanned caesareans, which may be the result of either undiagnosed breech presentation or planned vaginal breech delivery. Unplanned caesareans for breech presentation have been shown to be associated with increased maternal and neonatal morbidity compared with planned caesarean sections.^9^,^31^ Whereas the overall proportion of term pregnancies complicated by breech presentation does not warrant universal ultrasound screening,^32^ the increased risk of a recurrent breech presentation demonstrated in our study may justify ultrasound monitoring for identification of subsequent breech presentation for term pregnancies where there is a history of a breech-presenting infant.

Our study was limited to the examination of the risk factors identified and reliably reported on hospital and birth data. Although we reported birth defects as a dichotomous variable, we did not include specific defect diagnoses or associated complications, such as hydrocephalus, given the rarity of such specific diagnoses. Maternal prepregnancy body mass index (BMI) may be associated with breech occurrence and recurrence; however, NSW does not collect this item in birth data, and therefore it could not be included in these analyses. Although we report on mode of delivery at each breech presentation, it is worth noting that our study period straddles the period in which there was a major transition to caesarean section for breech presentation following the publication of results from the Term Breech Trial.^33^,^34^ This means that women having their second breech-presenting birth were at increased likelihood of having a caesarean birth because these women were more likely to be delivering later in the study period.

The strengths of our study include the use of longitudinally linked population-based data allowing us to follow the consecutive pregnancies of individual women, and the availability of validated data on risk factors and outcomes. By selecting term pregnancies we have avoided the effect of recurring preterm birth, and by conducting sensitivity analyses we have ruled out the possibility that we are reporting recurrence of birth defects or placenta praevia.

Importantly, our study presents occurrence and recurrence risks of breech presentation in an accessible format for patient counselling. That is, 4.2% of first pregnancies result in breech-presenting births, 9.9% of subsequent pregnancies will involve another breech presentation, and the rate after two prior breech deliveries rises to 27.5%. This represents a 3.2- and 13.9-fold increased risk of recurrent breech in a second and third pregnancy, respectively.

## Conclusion

In conclusion, women who have experienced a prior pregnancy with breech presentation at term are more likely than women without a prior breech history to give birth to a subsequent breech-presenting baby. These consistently elevated recurrence rates highlight the need for women with a history of breech delivery to be closely monitored in the latter stages of pregnancy.
